# Preparation of Glass-ceramics Using Chromium-containing Stainless Steel Slag: Crystal Structure and Solidification of Heavy Metal Chromium

**DOI:** 10.1038/s41598-018-37996-4

**Published:** 2019-02-13

**Authors:** ShunLi OuYang, YuXuan Zhang, YuXin Chen, ZengWu Zhao, Ming Wen, BaoWei Li, Yu Shi, MingZhe Zhang, ShiLiang Liu

**Affiliations:** Key Laboratory of Integrated Exploitation of Bayan Obo Multi-Metal Resources, Inner Mongolia University of Science and Technology, Baotou, 014010 China

## Abstract

It is a useful way to stabilize the elements of heavy metal in the glass-ceramics with the form of ions due to the environmental pollution of heavy metal, such as Cr. The glass-ceramics of excellent combination properties were prepared, and the effect of stabilizing Cr-containing stainless steel slag with different concentrations of nitric acid leaching test were investigated. It was found that the major crystalline phase was diopside or anorthite with or without the amount stainless steel slag. Moreover, the continuous refinement of grains exhibited with the increase of amount of stainless steel slag. The results indicated that the excellent physical and mechanical properties, including density (2.9 g/cm^3^), hardness (729.27HV0.3), bending strength (222.9 MPa), and the solid solution of Cr in excess of 0.00057% for the glass-ceramics were related to the change of microstructure and phase structure. There showed the potential for reusing and detoxifying stainless steel slag.

## Introduction

The accumulation of solid waste occupied a large amount of land and made the environmental pollution^1^. Importantly, the natural resources such as rivers, soil and groundwater were also polluted by the heavy metal elements^2–9^. Per ton of stainless steel slag, as a kind of solid waste produced during the production of stainless steel, were produced by three tons of stainless steel approximately^10^ to which the human body can be harmful through contaminated soil groundwater, especially the heavy metal element Cr^11,12^. The stainless steel slag is considered as high toxic waste because of the carcinogenic, mutagenic and toxic of the element Cr through dermal and oral exposure^13^. However, the byproduct stainless steel slag was continuously produced and accumulated due to the real demand of society for stainless steel. Therefore, it is urgent to apply a new method of focusing on the preparation of advanced materials using stainless steel slag as raw material and, at this time, the fact that reducing the environmental pollution by stabilizing the heavy metal element, such as Cr, in the crystal is a promising method. The glass-ceramics, as a new material, is composed of a crystalline phase and an amorphous phase so that it has good physical and chemical properties such as mechanics^14^, thermals^15^, optics^16^, electricity^17^, etc.

As so, the detoxifying treatment in it has been studied by many researchers performed preliminary research on reusing and recycling stainless steel slag. The working performance and mechanical properties of the cement were studied using it and the requirements of use application were met^18^. In addition, the non-toxic treatment of chromium in stainless steel slag was another important way to reduce the environmental pollution. The wet reduction method was used to reduce the content of exchangeable and carbonic acid bound chromium that was easily leached from the stainless steel slag. The remaining chromium was difficult to be leached^19^. Unfortunately, the pollution to the environment was caused by a large amount of accumulated pollutants again using this method due to the incomplete detoxification. Nevertheless, most of the heavy metal elements, Cr, Fe, etc. can be recovered as alloys with the high-temperature reduction method to detoxify the stainless steel slag by adding the appropriate amount of reducing agent^20,21^. And it was found that the method of solidification converted the toxic Cr (VI) into a stable Cr (III) and stabilized in the crystal lattice so as to the stainless steel slag was detoxified. As a result, the product can be reused as a building material with excellent performance^22^. Ma *et al*. sintered the stainless steel slag fully sintering, and Cr emitted very little in the emissions during this process within environmental limits^23^. Furthermore, the influence of the presence of chromium containing the different chemical valence state in the crystalline phase were leached by the ratio of crystallized glass on the solidification of chromium using Cr leaching ratio in glass-ceramics^8,11^ and, meanwhile, this is better way for studying the solidification mechanism of high performance glass-ceramics for chromium.

In view of the above facts, recycling stainless steel slag to obtain glass-ceramics was a kind of product with excellent performance being not harmful to the environment and human body. Thus, chromium-containing stainless steel slag to prepare glass-ceramics of CMAS system with high performance and, at the same time, curing Cr in the crystal structure were studied by the thermal behaviors, crystalline phase, grain shape and size. And furthermore, it is also expected that the relationship between the physical and mechanical properties of glass-ceramics and their microstructure feature and phase structure can be revealed.

## Methods

### Materials

Glass-ceramics were prepared from MgO, SiO_2_, CaO, Na_2_CO_3_, Na_2_B_4_O_7_, Al_2_O_3_, and Fe_2_O_3_ as the main raw material by melting. Afterwards, the mixed specimens with the increased of amount of the stainless steel slag containing 0 wt.%, 5 wt.%, 10 wt.%, 15 wt.%, 20 wt.% were prepared, respectively. The compositions of these specimens with increasing content of Cr_2_O_3_ in the stainless steel slag were shown in Table 1. In order to complete the nucleation and crystallization treatment, the five homogeneously mixed specimens were completely melted at a temperature of 1500 °C for 2 hours and then the melts were cast into the preheated stainless steel molds and, finally, were annealed at 600 °C for 3 h. After this, the glass specimens of annealing exhibited the glass-ceramics.

**Table 1:** Chemical composition of glass-ceramics.

Specimen	MgO	SiO_2_	CaO	Na_2_CO_3_	Na_2_B_4_O_7_	Al_2_O_3_	Fe_2_O_3_	Cr_2_O_3_
S1	8.5	47.0	16.9	5.9	7.7	8.5	5.6	0.0
S2	8.4	46.5	16.7	5.8	7.6	8.4	5.6	0.2
S3	8.3	46.1	16.6	5.7	7.5	8.3	5.5	0.5
S4	8.2	45.6	16.4	5.6	7.4	8.2	5.5	0.7
S5	8.1	45.1	16.3	5.5	7.4	8.1	5.4	0.9

### Testing method

The thermal behaviors of the glass specimens were examined by differential scanning calorimetry (DSC, STA449C, NETZSCH), using an empty Al_2_O_3_ crucible as a reference material and heating at 10 °C/min from room temperature to 1000 °C. The crystalline phase were determined by X-ray diffraction(XRD, X’pert Pro Powder, PANalytical) using Cu K ${\rm{\alpha }}$ radiation with a wavelength 0.1541 nm, in the 2-theta/° range from 10 to 80 degree, and at a step size of of 0.02° and a scan speed of 0.3 s/min of scanning rate at room temperature, which was operated at 40 KV and 40 mA. The XRD data was subjected to fit profile processing using X’Pert HighScore Plus (version: 3.0), and then the degree of crystallinity of the specimens was obtained by “Constant Background” value. The microstructure was obtained by filed emission scanning electron microscope(FESEM, SUPRA 55 FESEM, Carl Zeiss Jena) which is equipped with an Oxford EDS and HKL EBSD analysis system. The specimens were polished, corroded, and then sprayed with gold. The Raman spectrum system was constructed independently by our laboratory equipped with Andor Sham-rock SR-500i-C-R type Raman spectrometer and Andor iDus series water-air cooled CCD (Charge Coupled Device) detector product by UK ANDOR Company, and a 1200 grove/mm grating with a wavelength resolution about 0.05 nm. In the experiment, 50 times long focus objective lens (parameter: 50×/0.35) was used for focusing on the specimens, adjusting the specimen height to complete the focal point, focusing image collected by SunTime130E CMOS color digital camera, excited at the 355 nm line of semiconductor laser at which ~25 mW was output power each specimen. And then the Raman spectrometer was calibrated with a single crystal silicon prototype. All the signal data of each specimen was gathered at room temperature with 20 seconds exposure time, 5 times accumulation number, 20 s accumulation cycle time, and the scanning range was from 0 to 4000 cm^−1^. The densities were measured by Archimedes method. The three-point bending strength of rectified parallelepiped bars (3 mm × 4 mm × 40 mm) of glass-ceramics was tested by the CSS-88000 electronic universal testing machine. Each value was the average of five groups of data originated from the same specimens. Indentation experiments were conducted using a Vickers hardness tester (HV-SOA).

### Leaching Way

According to the standard “HJ/T 299–2007”, the long-term toxicity leaching test of the prepared glass-ceramics was performed using a mixed solution of sulfuric acid and nitric acid with a pH = 3.20 ± 0.05. The size of specimens was less than 75 mm and the liquid-solid ratio was 10:1. The content of chromium in the leach solution was distinguished by ICP-MS and compared to the standard specified in standard “GB 5085.3-2007” to evaluate the immobilization effect of glass-ceramics on chromium in the stainless steel slag.

In order to quantify the solidification effect of the prepared glass-ceramics on Cr element, a long-time leaching test was performed on the stainless steel slag and the glass-ceramics using a concentration of 5% nitric acid, respectively.

## Results and Discussion

### Differential thermal analysis

**Figure 1:**
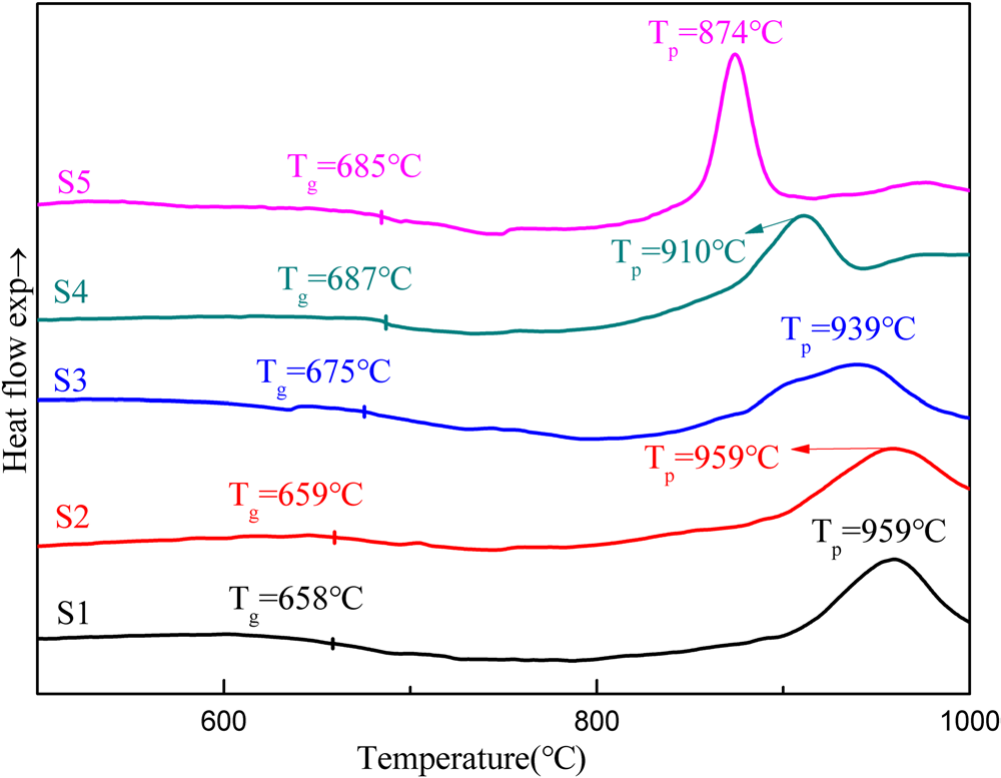
DSC curves of the glass with different contents of stainless steel slag.

DSC curves depict the thermal behaviors of five specimens, including transition temperature (T_g_) and peak temperature (T_p_), as shown in Fig. 1. With the addition of stainless steel slag, the glass T_g_ increases more slowly and around 670 °C while T_p_ decreases from 959 °C to 874 °C. At present, the conventional method by two-stage heat treatment was employed. The heat treatment with a low temperature that gives a high nucleation rate (around T_n_) was the first stage, and there exhibited a high density of nuclei throughout the interior of the glass. The growth of the nuclei at a reasonable rate shown in the second stage showed at a higher temperature around temperature T_g_^24^. The nucleation temperature was selected as 720 °C on the basis of T_n_ above the transition temperature of 50 °C^25^. According to the obvious exothermic peak, the crystallization temperatures of S1 to S5 were selected at 950 °C, 950 °C, 930 °C, 900 °C and 870 °C, and then held them for 2 hours, respectively.

### Phase analysis

**Figure 2:**
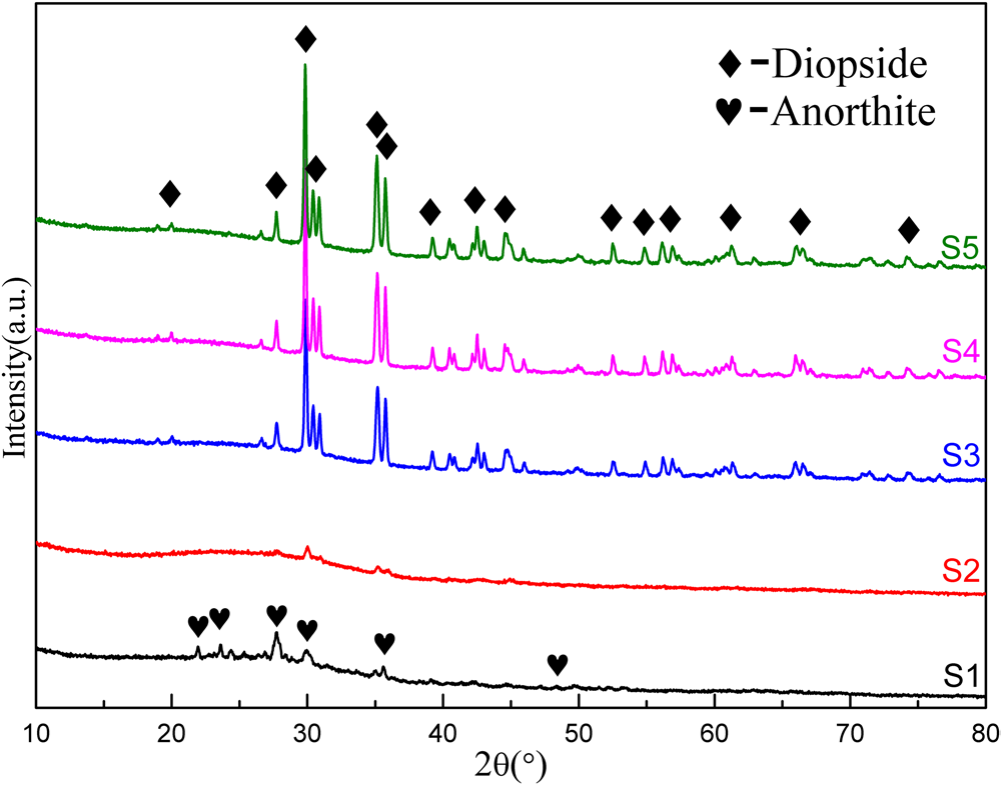
XRD patterns of glass-ceramics with different content of stainless steel slag.

The XRD patterns of crystalline phase are shown in Fig. 2. It is found that the major crystalline phase of glass-ceramics of CMAS system was transformed from anorthite to diopside. The major crystalline phase of specimen 1 is anorthite, (Ca, Na)(Si, Al)_4_O_8_ (${\rm{P}}\bar{1}$), while diopside, (Ca_0.78_Na_0.18_Mg_0.04_)(Mg_0.79_Fe_0.05_Cr_0.09_Al_0.07_)(Si_1.97_Al_0.03_O_6_) (C2/c), is the major crystalline phase in the other specimens with the increase of stainless steel slag containing chromium.

It was evident that the phase compositions of all the specimens were very similar except for the specimen 1 due to the absence of the element Cr. However, the crystalline phases produced by them were quite different. The major phase formed was anorthite without the addition of Cr and, to the contrary, was diopside with the amount of Cr_2_O_3_. The result indicated that Cr_2_O_3_ was a very good nucleating agent in the nucleation and growth of glass-ceramics owing to a strong electric field of Cr^3+^, which made the disordered phase transitions, namely glass structure, to the ordered phase transitions due to the phase separation, reduced the potential energy barrier for crystal growth. It is accordance with the previous research^26–28^. As for the Specimen 1, there was no nucleation agent if there was no the element Cr. Thus, the anorthite, as the major phase, appeared, including the elements Ca, Mg, etc., and the diopside disappeared due to the inhomogeneity of the matrix and air components during the process of growth.

### Micrographs analysis

**Figure 3:**
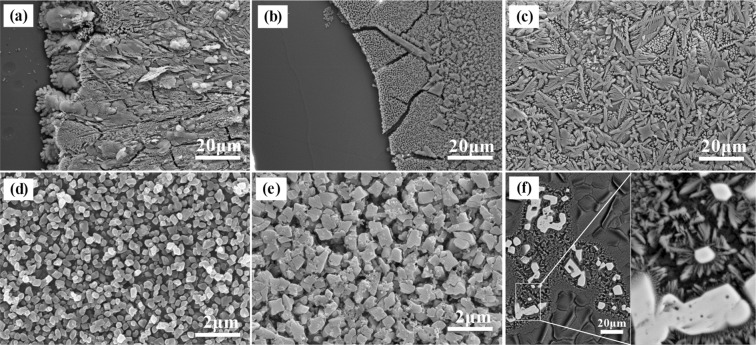
FESEM micrographs of glass-ceramics: (**a**) S1, (**b**) S2, (**c**) S3, (**d**) S4, (**e**) S5. The magnification of (**a**–**c**) is 1,000 times. The magnification of (**d**,**e**) is 10,000 times. The magnification of (**f**) is 500 times, and magnified the position of the spinel.

Figure 3 exhibits the morphology of glass-ceramics with the increase of stainless steel slag containing the amount of Cr_2_O_3_. The degree of crystallization of the specimens increased with the increasing stainless steel slag. The major phase of the S1 was anorthite and, moreover, the other four groups were diopside which is accordance with the results of XRD.

In the case of the S1, it contained a large amount of Ca, Al, Si etc. except for the element Cr. It was found that the crystalline grains grew from the surface to the inward and the crystallization occurred in the surface due to the inward proceeded by the small extent of crystalline phase, as shown in Fig. 3a. The formation of the anorthite in the phase boundary, caused by the heterogeneous nucleation, was the primary reason. Moreover, It should be noted that there was a clear boundary between the glass and the anorthite. For the other four groups of specimens, with a small amount of stainless steel slag added, the low content of Cr was enriched by its electric field so that it was not uniformly distributed throughout the S2, resulting in local crystallization, as shown in Fig. 3b. The particle size, near the glass phase, was greater than it in the interior owing to the less nucleating agents for which the crystalline grains to fully grow along the main zone axis and the secondary zone axis to form large dendritic grains were favorable. With the addition of stainless steel slag being up to 9.21 wt.%, the crystalline phase of S3 possessed the large particle size with the microstructure of dendrite, as shown in Fig. 3c, because of atomic migration and dendrite growth favored by the lower viscosity. Afterwards, there were many fine particles to replace the large grains with the content of stainless steel slag from 13.68 wt.% to 18.06 wt.% and, finally, the fine crystalline was evenly distributed, as shown in Fig. 3d,e. There existed limited growth on the crystalline due to the insufficient growth space so as to the fine grains formed.

**Figure 4:**
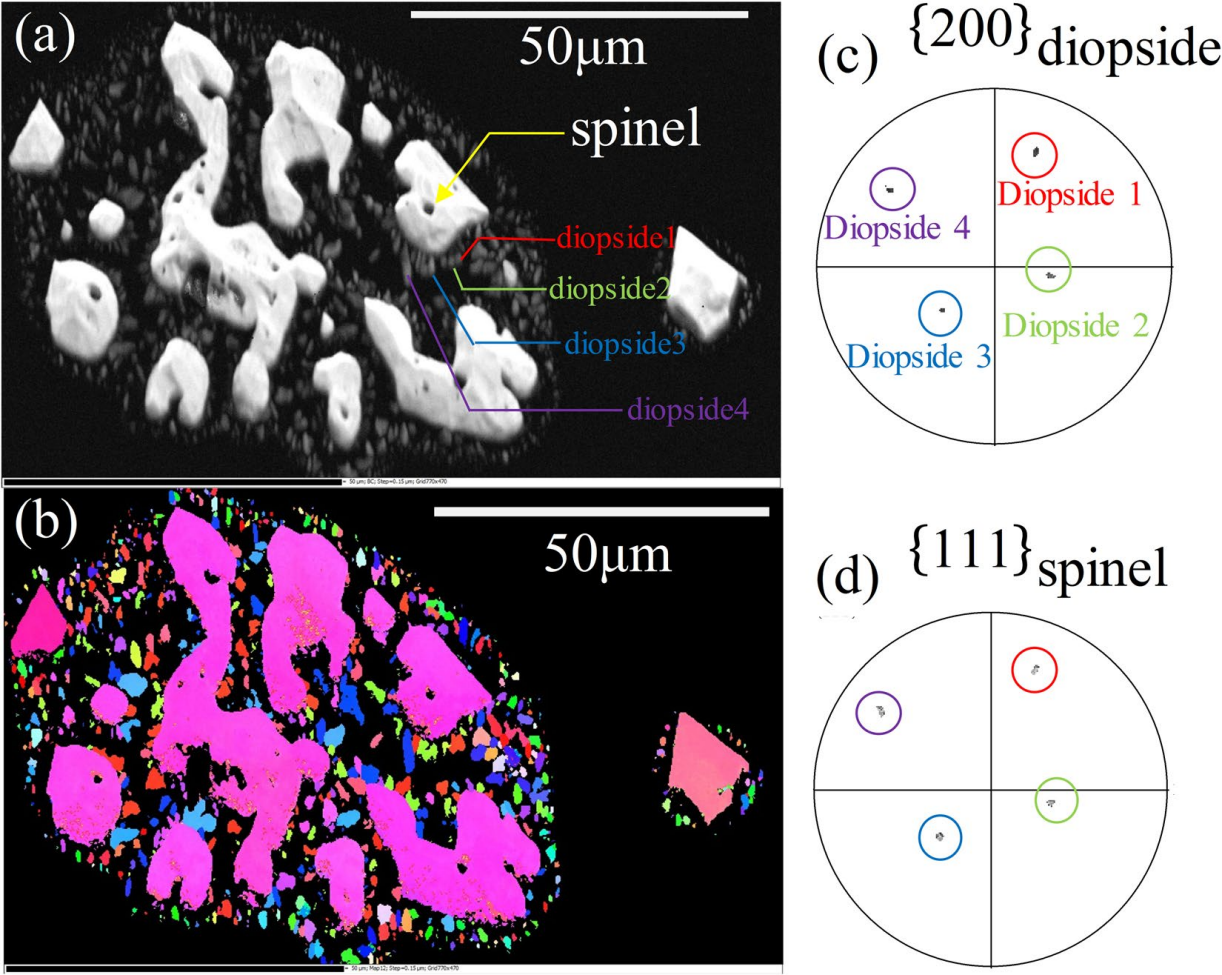
EBSD diagrams of the selected zones. (**a**) SEM image, (**b**) Grain-orientated mapping of selected zones, (**c**) Pole figure of the close-packed surface of diopside, (**d**) Pole figure of the close-packed surface of spinel.

Figure 4 shows the grain-orientated mapping and pole figure (PF) of electron backscattered diffraction (EBSD). As shown in Fig. 3f, the pink phase with spinel structure, as the minor phase, distinguished by EBSD, in the glass-ceramics specimens to which the stainless steel slag was added. The presence of the spinel structure resulted to the change of grain-orientated mapping, as shown in Fig. 4. There exhibited the crystalline phase and glass phase in the colored and black portions, respectively. And it can be seen that the phases, as the major phase, with different colors grown on the spinel phase, as a nucleation agent, as shown in Fig. 4a,b. And it can be proved by the orientation relationship by PF mapping, as shown in Fig. 4c,d, analyzing it by which the close-packed plane {200} of diopside and the close-packed plane {111} of spinel obtained. Besides, the different orientation relationship with PF mapping can be expressed with the different points overlapped, for instance, diopside one and spinel one as red circles were in the same position, diopside two and spinel two as green circles, and so on. So it can be considered that the presence of the spinel structure can induce the growth of the diopside structure. It is consistent with the previous results^29^.

**Figure 5:**
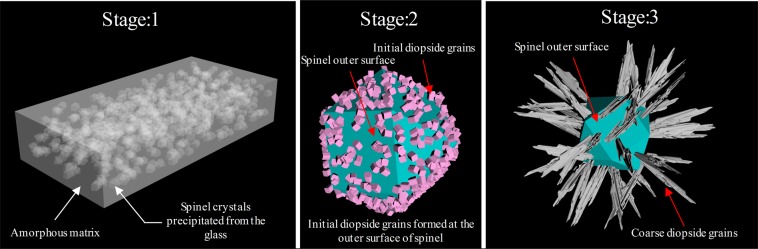
Schematic diagram of glass converted to glass-ceramics.

The process of converting the glass into the glass-ceramics with the additive stainless steel slag, divided into three stages, occurred during the heat treatment, as shown in Fig. 5. Firstly, a large quantity of crystalline with spinel structure formed in the glass specimens in the stage of nucleation, served as a heterogeneous nucleation zones for the crystallization. Furthermore, as the temperature increased, a great deal of small crystalline with diopside structure grew on the surface of the spinel. In the third stage, while the temperature reached above crystallization temperature and held for the period of time, the small crystalline with diopside structure grew up, and the entire interior of the specimens were filled with the crystalline phase eventually.

### Raman Spectroscopy

**Figure 6:**
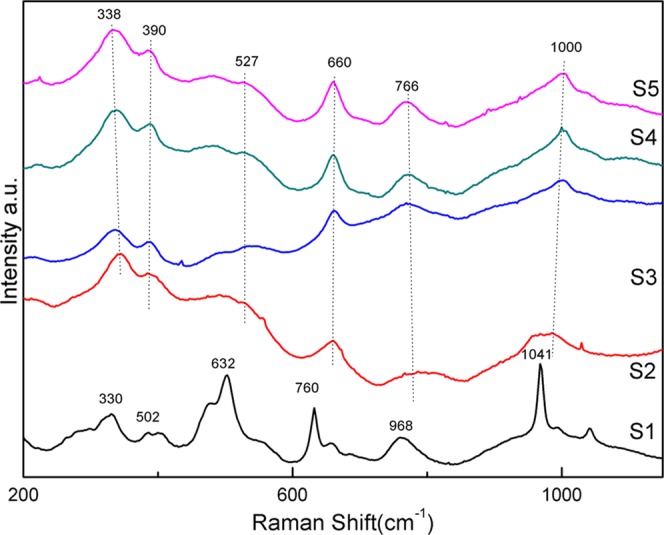
Raman spectra of the crystallization section of five groups specimens.

Figure 6 shows the results of Raman spectra of the glass-ceramics. In the specimen, S1, the strong bands intensity exhibited at 330 cm^−1^, 502 cm^−1^, 632 cm^−1^, 760 cm^−1^, 968 cm^−1^ and 1041 cm^−1^. However, there were a number of bands at 338 cm^−1^, 390 cm^−1^, 527 cm^−1^, 660 cm^−1^, 766 cm^−1^, 1000 cm^−1^, 1331 cm^−1^, and 1580 cm^−1^ among S2, S3, S4 and S5 that were close to the Raman spectral band, as shown in the assignment table of pyroxene^30^. It can be seen that the phase of S1 is not the same as that of the other four groups of specimens.

There existed the band at 338 cm^−1^ to be related to the bending vibration of alkali metals and oxygen (M-O) and the band at 390 cm^−1^ to be the deformation vibration of alkali metals and oxygen (M-O). The band at 527 cm^−1^ and 660 cm^−1^ was mostly associated with the bending vibration and symmetric bending vibrations of O-Si-O. The symmetrical stretching vibration of Si-O corresponded to the band at 1000 cm^−1^. In the amorphous phase, the stretching vibration of four non-bridge oxygen bonds was controlled by the peak with a wave number of 766 cm^−1^. The band 1331 cm^−1^ and 1581 cm^−1^ were assigned to the [BO_3_] group^31^, deteriorating the performance of the glass. The fact that governed by the content of the [BO_3_] group decreased with the increased content of chromium. This is of great significance for the excellent performance of glass-ceramics.

### Physical and mechanical properties of glass-ceramics

The density and degree of crystallization of the specimens increased with the addition of stainless steel slag apart from the bending strength, increasing primarily and then decreasing, as shown in Table 2. The low density, determined by the low degree of crystallization that was affected by the closeness of the structure accumulation^32^, were formatted in the S1 and S2, resulting in the glass phase mostly. As shown in Fig. 3a–e, the crystalline size of S5 was smaller and the packing was denser than the others so as to the density of S5 was the largest. Despite there were the microstructure of dendrite in the S3 and S4, as shown in Fig. 3c,d, the better properties were shown in S4 with small particle size than that of S3 possessed the large particle size. Besides, the degree of crystallization was the other important factor deciding hardness and bending strength. As shown in Table 2, the bending strength of S4 reached 222.9 Mpa. Evidently, it was larger than the others. Correspondingly, the high degree of the crystallization reached 7.30% and the fine branches of dendrite, deformed by external forces, were interlocked. Meanwhile, there was remaining glass phase, wrecked continuity of the crystalline phase, to be observed in the glass-ceramics, such as S1 and S2, indicating that the decreasing properties of the glass-ceramics were related to the increase of glass phase^32^. It has been proved that there was a diameter leads to maximum bending strength values, while smaller or larger diameters produce lower strength values^33^. Thus, the grain size of dendrite about the S3 was extremely large, resulting in the generation of crack, in which an external force was enforced, and the bending resistance was poor. As for S5, there exhibited the small crystalline size, being far away the optimum crystalline size, to result in the less bending strength compared to the S4.

**Table 2:** Mechanical properties of glass-ceramics with different stainless steel slag.

Specimen	Density (g/cm^3^)	Bending Strength (MPa)	Vickers Hardness (HV0.3)	Degree of Crystallization (%)
S1	2.51	117.4	599.49	3.69^*^
S2	2.59	132.1	665.04	2.47^*^
S3	2.84	141.7	711.58	6.60
S4	2.88	222.9	717.36	7.30
S5	2.9	204.7	729.27	8.13

The *in the upper right degree of crystallization data represents data for reference only. Since the specimen is non-uniformly crystallized, the data measured by XRD depends on the sampling position.

### Immobilization effect of heavy metals

After the leaching test of the glass-ceramics continued 18 hours, the concentration of Cr in the leachate was lower than 0.007 ppm as shown in Table 3, which was much lower than the 5 ppm required by the Chinese standard “GB 5085.3-2007”. It was found that the glass-ceramics showed excellent performance in the Cr curing after the 336 h leaching test and the increasing rate of concentration of Cr in the leachate became slowly. The results indicated that the crystalline phase with diopside structure formed with the addition of stainless steel slag. It was obvious that the oxide Cr_2_O_3_, as a nucleating agent, stabilizes in the crystalline phase. Since the atoms in the lattice were in equilibrium, resulting in a long-range order in the crystalline phase, the Gibbs free energy reached the minimum. Therefore, the element Cr stabilized the crystal structure was stably present in the crystalline phase because of the most stable crystalline state^34^. In addition, the crystalline phases were also surrounded by a layer of glass, as shown in Fig. 4, which prohibited the element Cr for leaching. It is accordance with the previous research^6,8^.

**Table 3:** Concentrations of Cr in leachate using Chinese standard “HJ/T 299–2007” (ppm).

Time(h)	S1(ppm)	S2(ppm)	S3(ppm)	S4(ppm)	S5(ppm)
9	~	<0.001	<0.001	<0.001	<0.001
18	~	0.001	<0.001	<0.001	<0.001
36	~	0.002	0.003	<0.001	0.001
60	~	0.001	0.003	0.002	0.001
96	~	0.005	0.003	0.001	0.001
168	~	0.006	0.003	0.001	0.002
240	~	0.006	0.004	0.002	0.001
336	~	0.007	0.005	0.002	0.003

“~” means that the specimen does not contain Cr and the concentration of Cr is not measured in the ICP-MS test.

In order to further quantify the solidification effect of the glass-ceramics on the chromium ions, leaching tests were performed on the stainless steel slag and the glass-ceramics using a nitric acid solution, a concentration of 5%, respectively. The results were shown in Table 4. It was evident that the content of chromium ions from the glass-ceramics by the leaching fell off hundreds and thousands of times, compared to the stainless steel slag. As the increased trend of logarithmic function got slowly, the leaching concentration of Cr will not be much more than the content of Cr, 40.36 ppm, in the leachate. In the case of 5% nitric acid solution, unstable Cr will leach into the leachate.

**Table 4:** Concentration of Cr in 5% Nitric Acid Leachate and Calculation of Data Processing.

Time(h)	SSS(ppm)	S1(ppm)	S2(ppm)	S3(ppm)	S4(ppm)	S5(ppm)
9	182.6	~	5.365	0.759	0.202	0.112
18	222.7	~	8.552	1.026	0.269	0.159
36	292.1	~	13.71	1.761	0.363	0.225
72	445.7	~	24.89	2.386	0.572	0.37
144	565.4	~	34.09	3.866	0.754	0.466
288	758.4	~	40.36	4.222	0.892	0.516
UR(%)			0.20180	0.008444	0.00127	0.00057

“~” means that the specimen does not contain Cr and the concentration of Cr was not measured in the ICP-MS test.

Therefore, an index, unstable ratio (UR), was introduced, which was expressed as the percentage of the mass of Cr in the leachate to the mass of Cr in the specimen. An equation for calculation of the UR was followed as:

$${\rm{UR}}=\frac{{\rm{Leachate}}\,{\rm{volume}}({\rm{L}})\times {\rm{Leachate}}\,\mathrm{concentration}(\mathrm{ppm})}{{\rm{Leaching}}\,{\rm{specimen}}\,{\rm{quality}}({\rm{g}})\times {\rm{Mass}}\,{\rm{percentage}}\,{\rm{of}}\,\mathrm{Cr}(\mathrm{wt}{\rm{.}}\, \% )}\times \mathrm{100} \% $$

It can be concluded that the degree of crystallinity increased while the percentage of element Cr decreased, indicating that most of the element Cr was cured in the glass-ceramics.

## Conclusions

The glass-ceramics with excellent properties were prepared with the addition of Chromium-containing Stainless Steel Slag, as the additive solid waste, and made the heavy metal element, Cr, solidified in the crystal lattice successfully. The results indicated that the crystalline size was continuously refined with the increased additive Stainless Steel Slag. The bending strength exhibited maximum, 222.9 MPA, due to the interlocking phenomenon, caused by the deformed fine branches of dendrite with the external forces, as the amount of Stainless Steel Slag being up to 13.68 wt.%. Afterwards, The hardness reached 721.27 HV in which the degree of crystallinity reached 8.13% as the content of Stainless Steel Slag was 18.06 wt.% and, meanwhile, the amount of chromium, 99.999%, stabilized in the crystal structure of glass-ceramics. This was because the major phase with diopside structure was formed on the basis of the minor phase with the spinel structure, as the nucleating agent of glass crystallization, with the addition of Chromium-containing Stainless Steel. So the environmental pollution for the heavy metal can be reduced by preparing the glass-ceramics using solid waste as raw materials.
